# 

**Published:** 1890-03

**Authors:** 


### Communications.

### MISSOURI STATE DENTAL ASSOCIATION.

MEXICO, MO., March 15th, 1890.

*Editor of the American Journal of Dental Science*,

Dear Doctor :— We wish to call your attention to the next meeting of the Missouri State Dental Association, which will be held at Pertle Springs, July 8, 9, 10, 11, 1890.

No effort will be spared to make this meeting one of the largest and most interesting in the history of the Association.

The American Dental Association will meet in Missouri next August, and it is epecially desirable that we have a large attendance at our next meeting so that we may make proper arrangements to receive the members of the American Dental Association in a manner that will reflect credit upon the dentists of Missouri.

Now is the time to make your plans so that you may be able to be with us, and we earnestly solicit your presence.

Fraternally yours,

J. F. McWILLIAMS,
W. L. REED,
W. H. BUCKLEY,
Ex. Committee.

### TENTH INTERNATIONAL MEDICAL CONGRESS, BERLIN.

SECTION XIV.—DISEASES OF THE TEETH.

*To Dr. F. J. S. Gorgas:*

In accordance with the decision of the Ninth Congress held in Washington in 1886, the Tenth International Congress will be held at Berlin, from the 4th to the 10th of August, 1890.

By the Committee of Organization at Berlin, the undersigned have been nominated as American officers of the Section, and they earnestly desire that such a delegation shall be present as shall fitly and honorably represent this Country. They therefore cordially invite your attendance at the Congress and desire that you will at the earliest moment notify the Honorary Secretary of your intention.

Those who wish to offer any communication to the Congress or who have anything of interest to present, should send a sufficient abstract of the paper, or a full description of the apparatus or device, to Prof. Dr. W. D. Miller, No. 32, West Voss Street, Berlin, Germany; or it may be sent to the Honorary Secretary, at Cambridge, Mass., for transmission to Berlin.

W. C. BARRETT, M.D., D.D.S., Buffalo, N. Y.
J. TAFT, M.D., D.D.S., Cincinnati, Ohio.
E. S. TALBOT, M.D., D.D.S., Chicago, Ill.
H. J. MCKELLOPS, D. D. S., St. Louis, Mo.
Honorary Presidents.

R. R. ANDREWS,
Honorary Secretary,
432 Harvard St., Cambridge, Mass.

### TWENTIETH ANNUAL MEETING OF THE SOUTH CAROLINA STATE DENTAL ASSOCIATION.

The twentieth annual meeting of the South Carolina State Dental Association, will be held in Charleston, commencing Tuesday, May 13th, 1890, at the Charleston Hotel, at 12 o'clock M.

The Association will be glad to hear from any visiting Dentist, either by voluntary essay or at a clinic, as opportunities for clinics will be furnished. Visitors from our neighboring states as well as Members of the profession at large, are cordially invited to attend. Recent graduates and those in the State who have not already joined us are urgently requested to be present and join the Association, thus aiding the older members in elevating the practice of dentistry in the State.

Every effort will be put forward to make this meeting truly successful and really interesting, and the President expresses the hope that each member will feel that the success of the meeting will depend upon his individual presence and assistance.

In the future, beginning with the coming session, let it not be said that a few men control the Association, but let all work together to make it a power in the State, thereby inducing its proper recognition by the State Authorities in enforcing the Dental Law. This can only be done by a full appreciation of its objects by the profession generally. "In union there is strength."

Members knowing any new men coming in the State to practice will oblige by forwarding names to the Secretary.

There will be a full line of Dental Goods for inspection including new inventions and improvements within the last year. Col. Selby of the S. S. White Dental Manufacturing Co. superintending the same.

The Charleston Hotel offers a Meeting and Clinic Rooms and makes a rate of $2.50 per day to those attending the Meeting. Lower rates can be obtained elsewhere.

Round trip rates on rail roads four cents per mile.

L. P. DOTTERER,

*Rec. Sect'y. S. C. S. D. A.*

### A CALL TO MEDICAL SCHOOLS.

*To the Medical Colleges of the United States:*

The following Baltimore Medical Schools, UNIVERSITY OF MARYLAND, BALTIMORE MEDICAL COLLEGE, COLLEGE OF PHYSICIANS AND SURGEONS, BALTIMORE UNIVERSITY, WOMAN'S MEDICAL COLLEGE OF BALTIMORE, and the STAFF OF THE JOHNS HOPKINS HOSPITAL, having met for the consideration of reforms urgently needed in the system of medical education hitherto in operation in this country, after a full discussion of this most important subject, have come to the conclusion that it is not expedient, nor indeed practicable for the medical schools of any State to assume *alone* the responsibility of adopting advanced methods. Yet fully convinced of the pressing need of a change and earnestly desirous ts see it consummated, they are unwilling to let matters rest longer as they are, without at least an effort on their part to improve them. They have determined, therefore, to issue this appeal to the medical schools of the United States for their co-operations in inaugurating a *national advance.* Fully aware of previous ineffectual efforts in this direction, they yet realize that times have greatly changed since these efforts were made, and they believe that a repetition of them at this time would have a good prospect of success. The approaching meeting of the American Medical Association, drawing delegates, as it will, from every part of the country, offers a good opportunity for convening those who are interested in the contemplated changes. We therefore invite you to join with us in holding a conference for the full consideration of "Medical Education in this Country and Measures for its Improvement," and we request that you will appoint, at your earliest convenience, one or more delegates from your Faculty to represent it at a meeting to be held at Nashville, Tennessee, on the 21st of May, 1890, at 3 P. M. It is requested that delegates should be instructed, as far as possible, as regards the wishes of their Faculties upon the various measures now proposed in connection with advances in medical instructions, in order that definite results may be arrived at with the least possible delay and trouble. The following subjects are considered as most likely to to come up for discussion :

1. Three Years Course of Six-Months' Sessions.
2. Graded Curriculum.
3. Written and Oral Examinations.
4. Preliminary Examination in English.
5. Laboratory Instruction in Chemistry, Histology and Pathology.

A. FRIEDENWALD, M. D., President.
EUGENE E. CORDELL, M.D.., Secretary.
*On behalf of the Baltimore Faculties.*

BALTIMORE, MARCH 20TH, 1890.

---

DISLOCATION INFERIOR MAXILLARY.—March 25, at 6 A. M, was hastily summoned by Mrs. E. Basledo to reduce laxation of inferior maxilla, caused by suddenly gaping. Reduced same without difficulty. The family of the aged lady were greatly frightened, at what appeared to them, a very formidable accident. After the extraction of a number of worthless teeth, we order the gums to be daily *brushed* with soap and a drop or two of oil of winter green to hasten absorption, harden and heal the gums. The *after* pains *disappeared* under such treatment. DR. BOWNE.

---

A VALUABLE REMEDY.—BY DR. BOWNE.—For violent toothache, dentists may depend upon the following combination for its marvelous and instantaneous effects. It is a remedy of unrivaled power and absolutely reliable. " Break a hypodermic tablet of ¼ grain morphine sulphate, et atropine sulphate 1-150 grain in four parts, dissolve one part in ten drops of *warm*, *well*, *spring* or *river* water thoroughly. A perfect solution of the partial tablet having been made, it is drawn up into the syringe and the contents thereof, slowly and cautiously injected into the hard gums surrounding the aching tooth. Several applications may be made until all of the contents of the syringe are injected. No danger of bad after-effects can result from the dose used, as it represents but 1-16 grain of morphine sulphate and 1-600 grain of the powerful alkaloid derived from belladonna, "atropine sulphate." "*Sulphate* of atropine is freely soluble in water, the pure atropine is not. Tablets of the above formula may be purchased of John Wyeth & Brother, of Philadelphia. They will prove an invaluable addition to the armamentarium of every progressive dentist." Always prepare a *fresh solution for every case.*

Kingston, N. J., March, 1890.

---

### Editorial, Etc.

### To Readers and Correspondents.

Communications intended for publication, and exchange journals and papers, should be addressed to the Editor of The American Journal of Dental Science, No. 845 N. Eutaw St. Baltimore, Md. All letters relating to the business of the Journal, or containing cash remittances, to be directed to Messrs. Snowden & Cowman. No. 9 W. Fayette Street, Baltimore, Md. Articles for publication should be received by the Editor at least two weeks previous to the first of the month on which the number in which it is designed for them to appear, is issued.

### CONTENTS:

☞ The American Journal of Dental Science will contain fifty pages of reading matter in each number, making a volume of about Six Hundred pages,

EXCLUSIVE OF ADVERTISEMENTS.

